# P-1895. Ending The Stigma: Role of an Interprofessional Training Program in Advancing Comprehensive HIV Care

**DOI:** 10.1093/ofid/ofaf695.2064

**Published:** 2026-01-11

**Authors:** Evelyn Villacorta Cari, Scot Mattingly, Nathalie Dietrich, Frank Romanelli, Alice C Thornton

**Affiliations:** University of Kentucky, Lexington, KY; University of Kentucky, Lexington, KY; University of Kentucky, Lexington, KY; University of Kentucky, Lexington, KY; The University of Kentucky, Lexington, Kentucky

## Abstract

**Background:**

Interprofessional training programs are essential for fostering collaboration across disciplines and developing integrated care models. This is particularly important for individuals receiving HIV care, who often encounter stigma that may prevent them from seeking medical services.

**Figure 1:**
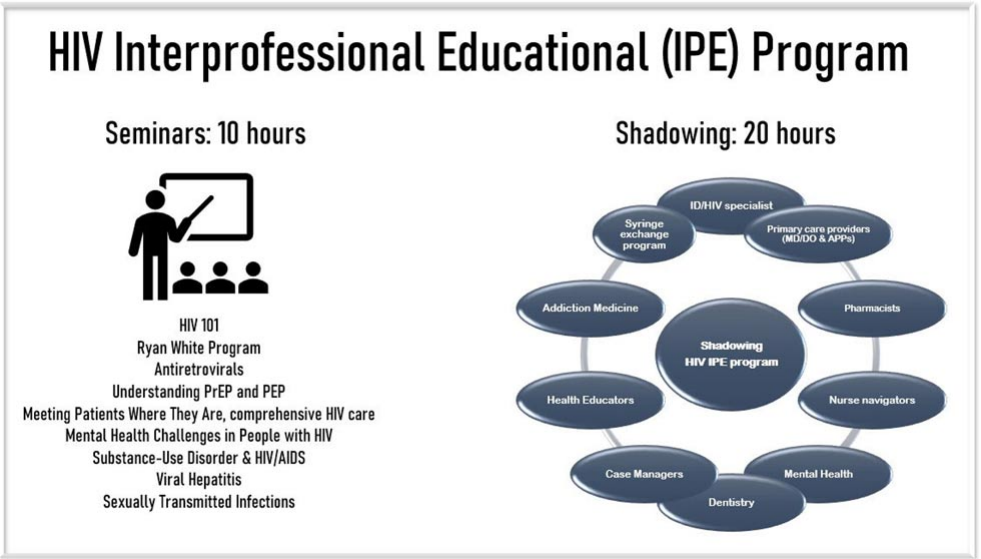
Design of the Interprofessional/comprehensive HIV training program

**Figure 2:**
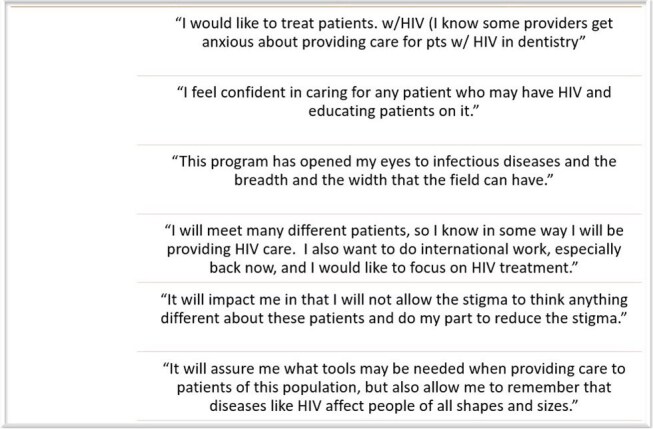
Self-reflection from HIV IPE students regarding the program's impact on their education

**Methods:**

Qualitative evaluation of students' feedback after completing an HIV IPE training program.

Students were selected to participate using a blinded review process. Applicants answered prompts regarding their motivation and fit for the program, including interest in HIV care, expected gains, and prior experience with diverse and underserved populations.

IPE students participated in 10 hours of didactic sessions and completed pre-session assignments to encourage interdisciplinary collaboration. Students also completed at least 20 hours of exposure to comprehensive HIV care with physicians, pharmacists, case managers/social workers, advanced practice providers, and mental health counselors. As part of an innovative approach, students also participated in activities serving marginalized populations, including the unhoused individuals at a local free clinic and individuals who inject drugs in the local syringe service program (Figure 1).

**Results:**

From January 2024 to May 2025, 14 students joined the program: pharmacy (five), medicine (three), nursing (three), dentistry (two), and physician assistant (one). Participant feedback revealed several key themes. Didactics were consistently described as engaging, informative, and relevant, with particular praise for sessions focused on PrEP/PEP, hepatitis, mental health, and Kentucky-specific HIV healthcare information. Shadowing experiences were also highly valued, providing students meaningful exposure to real-world interprofessional collaboration. Participants frequently highlighted the program’s well-organized structure, opportunities for professional networking, and broad exposure to diverse healthcare roles. Final self-reflection examples about HIV care are summarized in Figure 2.

**Conclusion:**

HIV IPE training programs can be utilized to improve HIV education of students from multiple health care disciplines with the ultimate goal of increasing a future informed workforce.

**Disclosures:**

All Authors: No reported disclosures

